# Thermal inactivation of African swine fever virus in feed ingredients

**DOI:** 10.1038/s41598-022-20290-9

**Published:** 2022-09-26

**Authors:** Tapanut Songkasupa, Prakit Boonpornprasert, Nutthakarn Suwankitwat, Walaiporn Lohlamoh, Chackrit Nuengjamnong, Suphachai Nuanualsuwan

**Affiliations:** 1 Virology Laboratory, National Institute of Animal Health, Chatuchak, Bangkok, Thailand; 2 Department of Animal Husbandry, Faculty of Veterinary Science, Chulalongkorn University, Bangkok, Thailand; 3 Department of Veterinary Public Health, Faculty of Veterinary Science, Chulalongkorn University, Bangkok, Thailand; 4 Center of Excellence for Food and Water Risk Analysis (FAWRA), Department of Veterinary Public Health, Faculty of Veterinary Science, Chulalongkorn University, Bangkok, Thailand

**Keywords:** Applied microbiology, Environmental microbiology, Pathogens, Virology

## Abstract

African swine fever virus (ASFV) causes a fatal infectious disease affecting domestic pigs and wild boars. ASFV is highly stable and easily transmitted by consumption of contaminated swine feed and pork products. Heat treatment of feed ingredients is a means to minimize the risk of contamination through swine feed consumption. The objectives of this study were to determine the thermal inactivation of ASFV in non-animal and animal origin feed ingredients. The rate of thermal inactivation is represented by decimal reduction time (*D*_T_) or time required to reduce ASFV per 1 log at temperature *T*. The mean *D*_60_, *D*_70_, *D*_80_ and *D*_85_ of meat and bone meal (MBM), soybean meal (SBM), and maize grain (MZ) are in the ranges 5.11–6.78, 2.19–3.01, 0.99–2.02, and 0.16–0.99 min, respectively. *D*_T_ is used to compare the heat resistance of ASFV in the feed ingredient matrices. The mean *D*_T_ of ASFV in MBM, SBM and MZ was not statistically significant, and the heat resistance of ASFV in MBM, SBM, and MZ was not different at 60, 70, 80, or 85 °C. The multiple *D*_T_ was used to develop a *D*_T_ model to predict *D*_T_ at various inactivation temperatures. The *D*_T_ models for MBM, SBM, and MZ are log *D*_T_ = − $\left( {\frac{T}{32.08}} \right)$ + 2.69, log *D*_T_ = − $\left( {\frac{T}{31.77}} \right)$ + 2.55, and log *D*_T_ = − $\left( {\frac{T}{18.96}} \right)$ + 4.01. To expand and ease the field applications, a spreadsheet predicting the *D*_T_ and the inactivation time (with 95% confidence interval) from these *D*_T_ models is available to download.

## Introduction

African swine fever virus (ASFV) causes a highly contagious viral disease of African and Eurasian wild boar and warthogs. The high morbidity and mortality rates of African swine fever (ASF) cause serious economic and production losses worldwide. The transmission routes are direct contact between sick and healthy pigs or indirect contact through contaminated fomites, feed, feed ingredients, pork products, or personnel^1^. ASFV belongs to the family *Asfarviridae* and the genus *Asfivirus*. ASFV is an enveloped double-stranded DNA arbovirus with a genome between 170 and 194 kbp in a virion diameter of 172–191 nm^2^. Even though by structure the ASFV is an enveloped virus, it is extremely stable in various conditions, the environment, and some animal products. The stability of ASFV in the environment causes outbreaks across continents. ASF outbreaks have been reported in many countries across the continents of Africa, Europe and Asia^3^.

The introduction of the porcine epidemic diarrhea virus (PEDV) into the North America in 2013 and 2014 occurred because of contaminated feed^4,5^. Feed biosecurity to minimize the risk of transboundary animal diseases has since become of major importance. Later on, the ASF outbreak in Latvia in 2014 demonstrated that non-animal origin feed could support the persistence of ASFV during shipment^6^. A previous report in 2019 demonstrated the identical genomes of ASFV from a pig and a dried blood pig feed sample in China^7^. Even though the scientific evidence regarding the stability of ASFV in feed is of significance to assess the potential risk and then formulate the appropriate risk management measure to mitigate the risk of introduction of ASFV of the importing countries, the number of studies regarding ASFV transmission through feed ingredients is limited.

In simulated trans-Pacific or trans-Atlantic transboundary models, ASFV was inoculated into various feed ingredients and complete feed to evaluate its residual infectivity^8^. The feed ingredients e.g. organic and conventional soybean meal and soy oil cake were inoculated with an ASFV titer of 5 log TCID_50_/ml. After traveling for 37 days in the trans-Pacific route from China to the United States with a mean temperature at 5 °C and 60–90% relative humidity, the residual titer of ASFV was determined using polymerase chain reaction and confirmed by swine bioassay. The residual titers of ASFV in the organic and conventional soybean meal, and soy oil cake were 3.0, 3.1 and 3.2 log TCID_50_/ml, respectively. The 1-log reductions of ASFV or the decimal reduction time (*D*_T_) at 5 °C, *D*_5_ of ASFV in the organic and conventional soybean meal, and soy oil cake were 18.5, 19.5, and 20.5 days, respectively, while the ASFV titers in blood, nasal fluid, and rectal fluid were as high as 6–8.7 log HAD_50_/ml, 1–4 log HAD_50_/ml, and 1–2 log HAD_50_/ml, respectively^9,10^. If the ASFV titer in feed ingredient is more than ASFV reduction during the transportation across country, then the risk of introduction of ASFV via contaminated feed is inevitable and risk management measures are mandatory.

Medium-chain fatty acid and formaldehyde-based feed additives served as a chemical additive to reduce the ASFV infectivity and the risk of ASFV transmission through feed ingredients^11^. A previous study developed a quantitative risk assessment to evaluate the probability of importing corn or soybean meal on ocean vessels contaminated with ASFV^12^. The *D*_T_ of ASFV from the thermal inactivation of corn and soybean meal was assumed from that of ASFV in pork serum^12^. Additionally, the effective thermal inactivation of ASFV at 56 °C for 70 min or 60 °C for 20 min was not specific to the feed ingredient^1^. The scientific evidence for ASFV inactivation by heat treatment of the feed ingredient was limited. Therefore, the objectives of this study were to assess the thermal inactivation of ASFV in non-animal and animal origin feed ingredients by heat processing temperatures and to develop a *D*_T_ model to predict *D*_T_ of at various inactivation temperatures.

## Materials and methods

### Cell preparation

Primary swine macrophages were aseptically collected from 24-week-old crossbred pigs in which the absence of PCV2, CSFV, PRRSV and ASFV by were confirmed by polymerase chain reaction assay (PCR). Peripheral blood morphonuclear cells (PBMCs) were prepared from defribrinated swine blood as previously described^1^. The cells were cultured in autogenous pig serum for maturation and then, after 3–4 days, monocyte-derived macrophage (MDM), that is macrophage-like round cells, were proliferated on a vessel surface. The cells were continually cultured in RPMI–1640 (Gibco, Waltham, MA, USA) culture medium containing 10% fetal bovine serum (Sigma–Aldrich, St. Louis, MO, USA) and supplemented with antibiotic–antimycotic solution (Gibco, Waltham, MA, USA).

### ASFV titration

The ASFV isolates (Asian epidemic strain, genotype II) were originated from pork products confiscated from international tourists during 2018 and 2020. The ASFV stocks (ASFV-NIAH-BL01–05) for the inactivation studies were routinely maintained and titrated in PBMCs culture and stored in aliquots at − 80 °C until use. All experiments with ASFV were performed at biosafety level 3 at the NIAH.

The viral titers of supernatants from each feed ingredient matrix spiked with ASFV isolates were determined by PBMC cell cultures. Approximately 1.5 × 10^6^ cells/well in 96-well plates were seeded in each well for 3–4 days prior to the assay. Fifty microliters of a tenfold serial solution of samples was inoculated into the wells in quadruplicate and incubated in the CO_2_ incubator at 37 °C for 5–7 days. The presence of haemadsorption (HAD) was examined under the microscope and the 50% HAD infectious dose per ml (HAD_50_/ml) was calculated using the Reed and Muench method^13^.

### Feed ingredients

Feed ingredients used in the study were meat and bone meal (MBM), soybean meal (SBM), and maize grain (MZ). The three feed ingredients were autoclaved to eliminate any possible ASFV contamination. The proximate analysis of feed ingredients was performed in triplicate at the Asia Medical and Agricultural Laboratory and Research Center to determine mean and standard deviation of crude protein (N × 6.25), total carbohydrate (excluding fiber), crude fiber, moisture, crude fat, and ash. The results are shown in Table 1.

**Table 1:** Proximate analysis of feed ingredients.

Composition	Percent (w/w)^a^		
	MBM	SBM	MZ
Crude protein	50.53 ± 3.51	48.75 ± 0.33	7.84 ± 0.27
Total carbohydrate	0	27.26 ± 0.50	71.72 ± 1.37
Crude fiber	1.26 ± 0.94	5.02 ± 0.33	3.22 ± 0.60
Moisture	5.15 ± 1.26	11.81 ± 0.37	12.92 ± 0.81
Crude fat	9.70 ± 1.07	1.04 ± 0.18	3.39 ± 0.33
Ash	33.38 ± 5.24	6.12 ± 0.24	0.91 ± 0.20

^a^Mean ± S.D. of three replicates.

### Thermal inactivation

One gram of ASFV-free feed ingredients was added to a 15 ml centrifuge tube. Prior to the addition of ASFV, the feed ingredients were preheated at 60, 70, 80, and 90 °C in a digitally controlled Heating Cooling Drybath (Thermo Fisher Scientific, Waltham, MA, USA). Then, the mixture was kept at the target temperture throughout the inactivation time. The initial infectious ASFV suspension had a titer of 5.0 log HAD_50_/ml. In this study, we prepared six samples per set per temperature. Each set consisted of triplicate samples spiked with 500 μl of ASFV; a positive control without any treatment (ASFV suspension), a positive non-treated control (feed ingredient sample spiked with ASFV) and a negative non-treated sample control (feed ingredient sample without ASFV, control at time zero). The inactivation temperature was monitored with a thermocouple. After the respective treatments, samples were immediately immersed in an ice bath for 30 min to stop the reaction. The samples were added and mixed with 0.5 ml of cell culture medium (RPMI–1640). The mixture was centrifuged, harvested, and stored at − 80 °C until the residual virus was titrated.

### Inactivation curve

The viral inactivation rate is assumed to follow first-order kinetics^14,15^. A linear inactivation curve is fitted to the reduction of log ASFV titer as a function of inactivation time at a constant inactivation temperature. The negative reciprocal of the slope of the inactivation curve is *D*_T_ as shown in the following equation:

1 $$\log N_{t} = - \frac{t}{{D_{t} }}\log N_{0}$$

Where, *N*_t_ and *N*_0_ are the ASFV titer at inactivation times *t* and zero, respectively.

### ***D***_T_ model

The DRT curve is derived from fitting multiple values of *D*_T_ on a semi-logarithmic scale across inactivation temperatures tested. The linear equation of the DRT curve is fitted to log *D*_T_ (DRT) as a function of inactivation temperature^16^. This linear equation is the *D*_T_ model. Analogous to *D*_T_, the *z* value is the negative reciprocal of the slope of the DRT curve. Therefore, the *z* value is the temperature required to change *D*_T_ by 90%. *D*_T_ of inactivation temperature could be predicted by the *z* value together with the *y*-intercept of the fitted linear equation as shown in the following equation:

2 $$\log D_{T} = - \frac{T}{z} + {\text{y-intercept}}$$

where,

*D*_T_ is the *D* of ASFV at inactivation temperatures *T.*

*z* is the were negative reciprocal of the slope.

### Statistical analysis

Regression analysis by an *F*-test was used to determine the statistical significance of the thermal inactivation curve and the DRT curve with a level of significance of 0.05 *i.e.* the slope of an inactivation curve as the regression coefficient of the inactivation-time variable (*x*-axis) is significantly different from zero (the heat treatment can actually lower the ASFV titer). Additionally, the slope of the DRT curve as the regression coefficient of the inactivation temperature variable (*x*-axis) is significantly different from zero, *i.e. D*_T_ is temperature dependent. The goodness-of-fit (*gof*) values of both the inactivation curve and the DRT curve were determined by the correlation coefficient (*r*^2^) and the root mean square error (RMSE)^17^. Two-way analysis of variance (ANOVA) with an interaction effect of inactivation temperature and feed ingredient was performed. Once ANOVA indicated a statistically significant difference, Tukey’s multiple comparison test was followed to determine the pair-wise *D*_T_ differences in terms of temperatures or feed ingredients. The IBM® SPSS® Statistics version 22 software (SPSS Inc., Chicago, IL, USA) was used to perform statistical analyses.

### Correlation of ingredient composition and ***D***_T_

The Pearson correlations coefficient (*r*) was used to evaluate the correlation of feed ingredient compositions and *D*_60_, *D*_70_, *D*_80_, and *D*_85_ of ASFV in inoculated ingredient.

### Ethical statement

Animal experiments regarding blood collection for the primary swine macrophages were performed under animal biosafety level 2 at the National Institute of Animal Health (NIAH), Bangkok, Thailand. All procedures were carried out in compliance with the Animal for Scientific Purpose Act 2015 (B.C. 2558). The ARRIVE guidelines 2.0 were followed for the care and use of laboratory animals. The animal study was reviewed and approved by the Institutional Animal Care and Use Committee at NIAH (Approval number EA-009/64(R)).

## Results

### ASFV survival of the heat treatment

**Figure 1:**
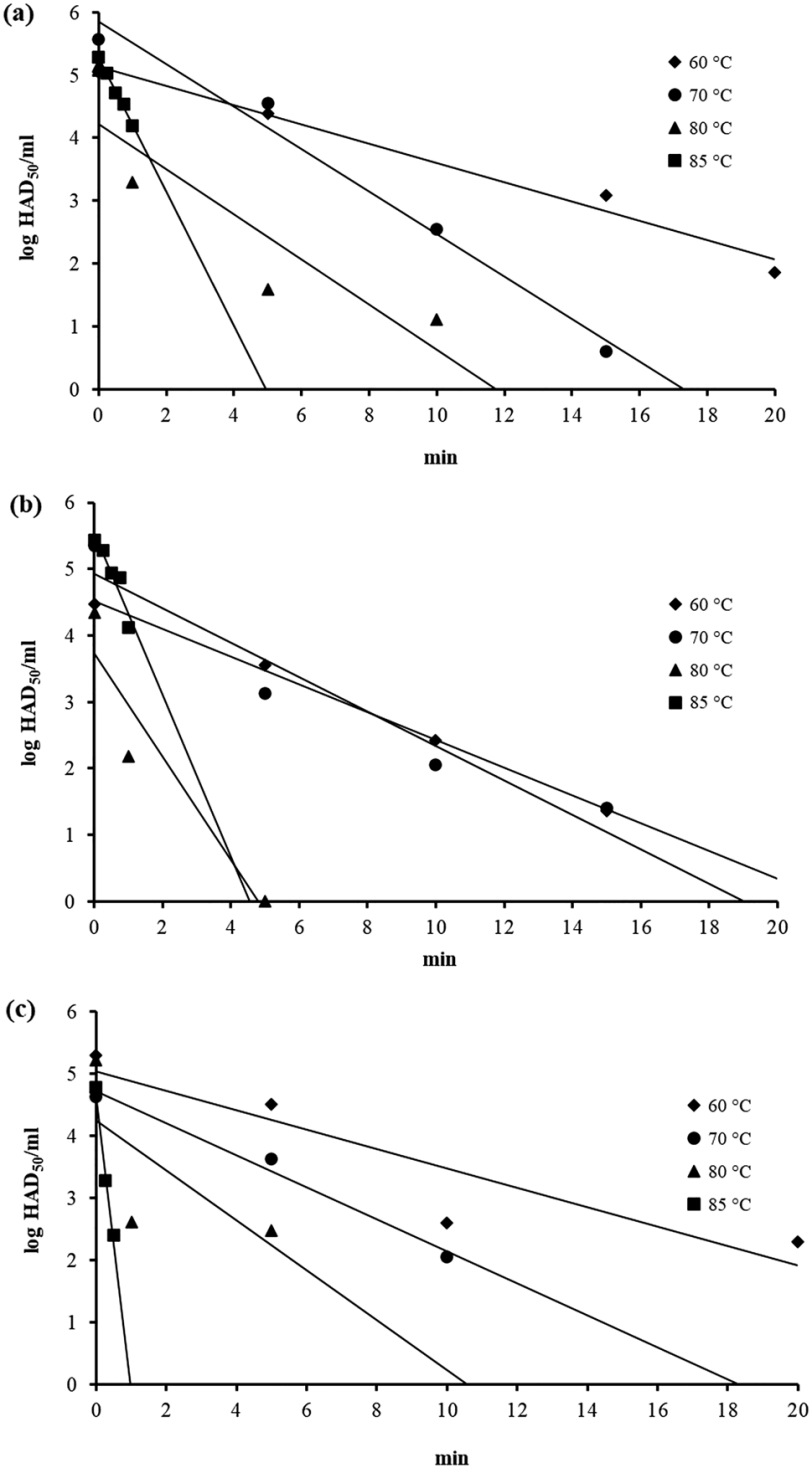
Thermal inactivation of ASFV at 60, 70, 80, and 85 °C in MBM **a** SBM **b** MZ **c.**

The feed ingredients inoculated with ASFV suspension in a centrifuge tube were subjected to heat treatment at 60, 70, 80, and 85 °C. The mean initial titers of ASFV in the feed ingredients were approximately 4–5 log HAD_50_/ml in conjunction with the appropriate inactivation time interval; this would allow stepwise reduction of ASFV titers throughout the experiment. The ASFV in the feed ingredient is sensitive to the heat treatment as the ASFV titers decrease along the inactivation time. The virucidal effect of heat treatment against ASFV in feed ingredients is as low as 60 °C. The ASFV titers drop faster at the higher temperature of heat treatment in all feed ingredients in this study (Fig. 1).

### ***D***_T_ of ASFV from thermal inactivation

**Figure 2:**
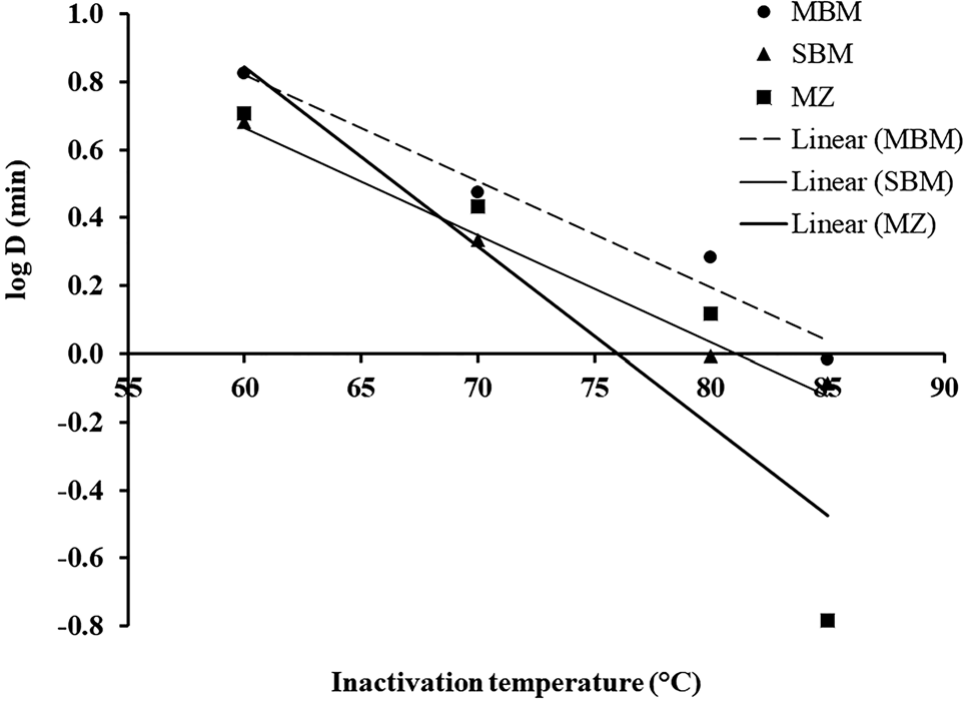
DRT curves were fitted to the log *D*_T_ of ASFV in feed ingredients.

The inactivation curve was calculated by fitting the linear regression to the log reduction of ASFV titer (*N*_t_) as inactivation time (*t*) increases. The thermal inactivation rate of ASFV was derived from the slope of this inactivation curve, which is the regression coefficient of the inactivation time variable (*x*-axis). The best-fit slope of the inactivation curve was always negative since the ASFV titers (*y*-axis) supposedly decrease along the inactivation time; therefore, indicating the virucidal activity of the heat treatment (Fig. 1). The mean *D*_T_, inactivation curves, and *gof* of ASFV in three feed ingredients across four inactivation temperatures are shown in Table 2. The mean *D*_60_, *D*_70_, *D*_80_, and *D*_85_ of all feed ingredients are in the ranges 5.11–6.78, 2.19–3.01, 0.99–2.02, and 0.16–0.99 min, respectively. Interaction effect of inactivation temperature and feed ingredient was significant (*p *≈ 4.1 × 10^−6^) (Table 3 and Fig. 2). For simple effect of inactivation temperature, the ASFV inactivation curves across four inactivation temperatures in 3 feed ingredients are statistically significant (*p* < 0.05). Therefore, in this study, the heat treatment at least 60 °C had virucidal activity against ASFV in the MBM, SBM, and MZ (Table 3).

**Table 2:** *D*_T_ and inactivation curves of ASFV in feed ingredients at various temperatures.

Feed ingredient	Temp. (°C)	*D*_T_ (min)^a^	Inactivation curve^b^	*gof*^c^		*p* value
				*r*^2^	RMSE	
MBM	60	6.78 ± 1.32	log *N*_*t*_ = − 0.15*t* + 5.11	0.83	0.67	< 0.001
	70	3.01 ± 0.56	log *N*_*t*_ = − 0.33*t* + 5.75	0.93	0.59	< 0.001
	80	2.02 ± 0.65	log *N*_*t*_ = − 0.49*t* + 4.35	0.55	1.47	0.013
	85	0.99 ± 0.27	*log N*_*t*_ = − 1.01*t* + 5.28	0.71	0.26	< 0.001
SBM	60	5.12 ± 2.28	log *N*_*t*_ = − 0.20*t* + 4.29	0.84	0.71	< 0.001
	70	2.19 ± 0.49	log *N*_*t*_ = − 0.46*t* + 5.18	0.89	0.77	< 0.001
	80	0.99 ± 0.07	log *N*_*t*_ = − 1.01*t* + 3.94	0.83	1.00	0.004
	85	0.82 ± 0.04	log *N*_*t*_ = − 1.21*t* + 5.54	0.83	0.21	< 0.001
MZ	60	5.11 ± 0.06	log *N*_*t*_ = − 0.20*t* + 5.20	0.70	1.03	< 0.001
	70	2.76 ± 0.62	log *N*_*t*_ = − 0.36*t* + 4.84	0.85	0.81	< 0.001
	80	1.38 ± 0.53	log *N*_*t*_ = − 0.72*t* + 5.94	0.50	2.55	0.03
	85	0.16 ± 0.01	log *N*_*t*_ = − 6.06*t* + 4.94	0.95	0.44	< 0.001

^a^Mean ± S.D. of three replicates.

^b^ASFV titer (log *N*_t_) at inactivation time *t* (min).

^c^Goodness-of-fit.

**Table 3:** Comparing the mean *D*_T_ (min) of ASFV in 3 feed ingredients of 4 inactivation temperatures.

Two-way ANOVA with interaction		Inactivation temperature (°C)				
		60	70	80	85	*F-*test (*p* value)
Feed ingredient	MBM	6.78^A,a^	3.01^A,b^	2.02^A,b,c^	0.99^A,c^	147.8 (1.3 × 10^−15^) *df* = 3
	SMB	5.12^A,a^	2.19^A,a^	0.99^A,b^	0.82^A,c^	
	MZ	5.11^A,a^	2.76^A,b^	1.38^A,c^	0.16^B,d^	
	*F-*test (*p* value)	19.5 (9.4 × 10^−6^) *df* = 2				11.7 (4.1 × 10^−6^)* *df* = 6

In the column-wise comparison, mean *D*_T_ with different letters implies that there are statistically significant differences (*p* < 0.05) among the different feed ingredients for the same inactivation temperature. (letters A and B). In the row-wise comparison, mean *D*_T_ with different letters implies that there are statistically significant differences (*p* < 0.05) among the different inactivation temperatures for the same feed ingredient (letters a through d).

*Interaction effect of inactivation temperature and feed ingredient.

The results of Tukey’s multiple comparisons of *D*_T_ in feed ingredients at 4 inactivation temperatures are shown in Table 3. Overall, the inactivation temperatures are negatively correlated with *D*_T_; as the inactivation temperature increases, the mean *D* decreases. In terms of temperature effect, the mean *D*_60_ of ASFV in all feed ingredients is highest and this is followed by mean *D*_70_, *D*_80_, and *D*_85_, respectively (*p* > 0.05) *i.e.* a higher inactivation temperature possesses a lower *D*_T_ and vice versa. The significant differences of *D*_T_ across inactivation temperature indicated the temperature effect. In terms of ingredient effect, the mean *D*_60_, *D*_70_, *D*_80_, and *D*_85_ of ASFV in MBM appeared to be highest; the significant interaction effect indicated that *D*_T_ in feed ingredient is influenced by inactivation temperature (Fig. 2). The mean *D*_60_, *D*_70_, and *D*_80_ of all feed ingredients were closely clustered together except *D*_85_. Furthermore, the two-way ANOVA with interaction effect of inactivation temperature and feed ingredient was rerun without *D*_85_ and the interaction effect was no longer significant (*p *≈ 0.74). According to Table 3, the mean *D*_T_ (except *D*_85_) of all feed ingredients were not statistically different (*p* > 0.05) as shown in Fig. 2. The thermal inactivation of ASFV in feed ingredient between 60–80 °C was independent upon type of feed ingredient tested in this study.

### ***D***_T_ model

Based on the mean *D*_T_ in Table 2, the DRT curves were drawn from the logarithmic *D*_T_ of ASFV in feed ingredients as *y*-axis versus the inactivation temperatures as *x*-axis (Fig. 2). The negative reciprocal of the slope of the DRT curve is defined as the *z* value. The mean and 95% CI of *z* values and the predicted *D*_T_ models of feed ingredients are shown in Table 4. The *gof* of all predicted *D*_T_ models indicates that the *D*_T_ models could well describe the log *D*_T_ by the inactivation temperatures.

**Table 4:** *Z* value and *D*_T_ model of ASFV in 3 feed ingredients.

Feed ingredient	*z* value (°C)		*D*_T_ model^a^	*gof*		*p* value
	Mean	95% CI		*r*^2^	RMSE	
MBM	32.08	25.55–41.30	log *D*_T_ = − $\left( {\frac{T}{32.08}} \right)$ + 2.69	0.88	0.12	< 0.001
SBM	31.77	26.09–40.63	log *D*_T_ = − $\left( {\frac{T}{31.77}} \right)$ + 2.55	0.91	0.10	< 0.001
MZ	18.96	13.97–29.50	log *D*_T_ = − $\left( {\frac{T}{18.96}} \right)$ + 4.01	0.80	0.28	< 0.001

^a^log *D*_T_ (min) for the unknown inactivation temperature *T* (ׄ°C).

### Correlations of feed ingredient compositions and ***D***_T_

Pearson correlation coefficients (*r*) of feed ingredient composition with crude protein, total carbohydrate, crude fiber, moisture, crude fat, and ash across *D*_60_, *D*_70_, *D*_75_, and *D*_80_ of ASFV are shown in Table 5. The *r* of crude protein and *D*_60_, *D*_70_, *D*_75_, and *D*_80_ were not consistent and were not further examined. The *r* of total carbohydrate and crude fiber were negative and inconsistent with *D*_60_, *D*_70_, *D*_75_, and *D*_80_. The moisture of feed ingredient and *D*_T_ were negatively correlated at a moderate level while crude fat and ash and *D*_T_ were positively correlated at a moderate level of correlation.

**Table 5:** Pearson correlation’s coefficients (*r*) of feed ingredient compositions and *D*_T_ of ASFV in feed ingredients.

Ingredient composition	Correlation coefficient (*r*)			
	*D*_60_	*D*_70_	*D*_75_	*D*_80_
Crude protein	0.29	− 0.11	0.11	0.93
Total carbohydrate	− 0.42	− 0.09	− 0.36	− 0.92
Crude fiber	− 0.47	− 0.58	− 0.73	− 0.20
Moisture	− 0.53	− 0.38	− 0.63	− 0.71
Crude fat	0.51	0.53	0.73	0.41
Ash	0.53	0.37	0.63	0.72

## Discussion

Since the porcine epidemic diarrhea virus (PEDV) has been established and spread in North America in 2013 and 2014, scientific evidence suggested the potential source of the virus as contaminated feed and feed ingredients^4,5^. It was evident that feed ingredients e.g. organic and conventional soybean meal and soy oil cake could retain ASFV infectivity in the trans-Pacific or trans-Atlantic shipment models with as low as 2–log reduction of ASFV at the average temperature of 5 °C with 60–90% relative humidity^8^. Additionally, ASF was reported throughout the region of Asia and Europe^3^. Therefore, the thermal inactivation ASFV in feed ingredients from affected countries as a risk management measure could potentially reduce a risk of the introduction of ASF to the importing country.

Some chemical agents have been demonstrated to have ASFV virucidal activity. The ASFV infectivities were significantly reduced < 2 log TCID_50_/ml after exposure to 0.07% caprylic acid (C_8_), 0.09% capric acid (C_10_), 0.10% lauric acid (C_12_), and 0.14% glycerol monolaurate (GM) in a suspension test at 37 °C for 60 min^18^. An aqueous formaldehyde-based additive at 0.03% and 0.3% inactivated ASFV titer 0.8 and 3.5 log TCID_50_/ml, respectively, at room temperature in 30-min inactivation time^11^. In the best-case scenario, the virucidal activity during the transoceanic shipment and the chemical agents would be combined; however, such ASFV titer reduction would be expected to be lower than the ASFV titer in the swine excretion^9,10^. Therefore, there is still a certain likelihood of residual infectivity of ASFV in the feed ingredient and this could lead to a risk for feed biosecurity and an outbreak of ASF in the importing country.

A previous study reported ASFV virucidal activity during the storage of field crops at 20 °C^19^. After 2 h of drying at 20 °C to simulate transport, wheat, barley, rye, triticale, corn, and peas were subjected to 1 h incubation at moderate inactivation temperatures between 40 and 75 °C. The ASFV inoculum on this field crop was infectious blood with a titer of 10^6^ HAD_50_/ml. Unfortunately, the ASFV titer by HAD test in all field crops was not detectable only after 2 h incubation at room temperature; therefore, the evidence of the moderate- or high-temperature heat treatment of ASFV in the field crop is not available^19^. Therefore, the result of this study could fill the research gap regarding the thermal inactivation of ASFV in non-animal and animal origin feed ingredients by the heat processing temperatures.

The ASFV suspension in this study had a titer of 6 log HAD_50_/ml. However, the recovery ASFV titer in the feed ingredient was approximately 4–5 log HAD_50_/ml. The reduced ASFV titer, after inoculating the feed ingredient, might be a result of the different hygroscopic properties of the feed ingredients^19^. The titer reduction of ASFV from drying was shown on some surface materials e.g. wood, steel, and plastic^20,21^. Even though the titer of ASFV was not completely recovered, the initial titers were still high enough to follow the stepwise reduction of ASFV titers throughout the experiment and significantly fit the inactivation curves (Fig. 1).

Even though the ASFV titers in all feed ingredients dropped as a function of inactivation time, the rates of inactivation of ASFV are a function of inactivation temperatures (Fig. 1). The ASFV titer at a higher inactivation temperature dropped faster than that at a lower inactivation temperature. In this study, the thermal inactivation rate was represented by *D*_T_. The *D*_T_ of foot–and–mouth disease viruses inactivated at a higher temperature was smaller than *D*_T_ inactivated at a lower temperature^17^. The mean *D*_T_ of ASFV in all feed ingredients across 4 inactivation temperatures in this study (Fig. 2) also followed the similar findings of this previous study.

Furthermore, *D*_T_ has been used to compare the inactivation rate of viruses in various feed ingredient matrices^22^. At a constant inactivation temperature, the higher *D*_T_ requires more time to inactivate the same virus titer and then is more heat resistant than the lower *D*_T_. In this study, ASFV in MBM appears to be the most heat resistant since the mean *D*_T_ of ASFV in MBM is highest except *D*_85_ (Table 3 and Fig. 2). This is in line with the significant interaction of inactivation temperature and feed ingredient and indicates that feed ingredient effect is influenced by the inactivation temperature. Therefore, two-way ANOVA with an interaction effect was rerun without *D*_85_ of all feed ingredients. The interaction of inactivation temperature (60, 70, and 80 °C) and feed ingredient became not significant (*p *≈ 0.74). Additionally, the mean *D*_T_ of MBM, SBM, and MZ of were not statistically significant (Table 3), so the heat resistance of ASFV in all feed ingredients between 60–80 °C is not different.

Even though the statistical analysis of *D*_T_ of ASFV indicated that the type of feed ingredient did not influence the ASFV heat resistance regardless of the inactivation temperature, it is noteworthy to further investigate how the ASFV heat resistance was affected by the nutritive composition of feed ingredients. According to the Pearson correlation coefficients (*r*) of nutritive composition of feed ingredients across *D*_T_ in Table 5, the moisture of feed ingredients and *D*_T_ were negatively correlated at a moderate level. This indicated that lower moisture might be more supportive for ASFV persistence in the feed ingredients. However, this finding was opposite to the result from a previous study where the transmissible gastroenteritis virus and the porcine epidemic diarrhea virus was found to be less heat resistant in dry feed ingredients^22^. Nevertheless, the additional analysis of the correlation of nutritive composition of feed ingredients and *D*_T_ of ASFV in this study also indicated that increased crude fat and ash may prolong ASFV survival during the heat treatment of feed ingredients.

For a similar thermal inactivation study with a different virus, *D*_T_ values of PEDV in feed ingredients were reported^23^. The *D*_60_, *D*_70_, and *D*_80_ of PEDV including ASFV were compared and are shown in Table 6. At 60 °C, PEDV appeared to be less heat resistant than ASFV in all feed ingredients. At 70 °C, PEDV appeared to be less heat resistant than ASFV only in MBM and SBM. At 80 °C, PEDV appeared to be more heat resistant than ASFV in all feed ingredients. However, according to the 95% confidence interval of *D*_T_ values of both viruses at 60, 70, and 80 °C, the *D*_T_ values of both viruses were not statistically different except for *D*_70_ in MBM. Therefore, the heat resistances of PEDV and ASFV in MBM, SBM, and MZ were not different except in MBM at 70 °C.

**Table 6:** Comparing *D*_T_ (min) of PEDV and ASFV in feed ingredient at 60, 70, and 80 °C

Feed ingredient	Inactivation temperature					
	60 °C		70 °C		80 °C	
	PEDV	ASFV	PEDV	ASFV	PEDV	ASFV
MBM	6.0^a^ (4.00–8.00)	6.44 (4.97–9.15)	2.4 (2.29–2.51)	3.15 (2.67–3.83)	2.3 (1.50–3.10)	2.55 (1.49–9.49)
SBM	3.3 (1.46–5.14)	5.70 (4.41–8.03)	1.3 (− 2.70–5.30)	2.46 (1.92–3.43)	1.7 (0.26–3.14)	1.13 (0.75–2.34)
MZ	3.4 (0.84–5.96)	5.11 (3.5–9.44)	3.3 (− 0.30–6.90)	2.79 (2.08–4.21)	2.2 (1.00–3.40)	0.91 (0.48–8.25)

^a^mean (95% confidence interval).

In this study, the thermal inactivations of ASFV in feed ingredients were performed at 60, 70, 80, and 85 °C. However, these inactivation temperatures might not be used as the processing temperature of choice in the swine feed industry. Therefore, the *D*_T_ model is developed to predict *D*_T_ values of some other inactivation temperatures (Table 4). For illustration purposes, the *D*_75_ of ASFV in MBM was predicted to be $10^{-\left( {\frac{75}{32.08}} \right)+2.69}$ or 2.2 min according to the *D*_T_ model of MPM in Table 4. As expected, *D*_75_ (2.2 min) of ASFV in MBM lies between *D*_70_ (3.01 min) and *D*_80_ (2.02 min). Interestingly, the concept of the predicted *D*_T_ model could be used to extend the scope of application for a certain study where a series of *D*_T_ values of a certain virus in the same feed ingredient was available. For example, a previous study demonstrated that a series of log *D*_60_, *D*_70_, *D*_80_, and *D*_90_ of PEDV in MBM were 0.78, 0.38, 0.36, and − 0.05 min, respectively (Table 6)^23^. Upon applying Eq. (2), the predicted linear *D*_T_ model of PED in MPM was log *D*_T_ = − $\left( \frac{T}{40.16} \right)$ = + 24 with a correlation coefficient (*r*^2^) of 0.91 (*p* < 0.05). Therefore, this linear *D*_*T*_ model is now able to predict *D*_T_ of PEDV in MBM of other inactivation temperatures than the original tested inactivation temperature in the original study.

The scope of prediction of the *D*_T_ model should be used with caution. Even though the *D*_T_ models were significantly fitted within the range of inactivation temperatures of this study (*p* < 0.05), the extrapolation of *D*_T_ should be restricted; since the *D*_T_ outside such a range of inactivation temperatures might not always be log-linear^23^. The non–linear trend of (log) *D*_T_ of PEDV in SBM was demonstrated in a previous study^23^. Log *D*_60_, *D*_70_, *D*_80_, and *D*_90_ of PEDV in SBM were 0.5, 0.1, 0.2, and 0.3 min, respectively (Table 6). The series of log *D*_T_ had a drastic drop of log *D*_70_ and a gradual rise from log *D*_80_ to log *D*_90_. This is an extreme example of a non-linear trend of *D*_*T*_. However fitting DRT curve is recommended to smooth out the outlier or uncertainty of *D*_T_. In the case of underestimating the inactivation time, this residual ASFV titer could present a risk for feed biosecurity. Therefore, the restricted use of *D*_T_ model to predict *D*_T_ of the unknown inactivation temperature could never be overemphasized.

Since the *D*_T_ models against ASFV in all feed ingredients demonstrated in this study are complicated and prone to error, an easy spreadsheet predicting the *D*_T_ and the inactivation time from these *D*_T_ models is provided. This encoded spreadsheet is intended to expand and ease the field applications by simply entering the inactivation temperatures including desire log reduction; this spreadsheet instantly provide the lower and upper 95% confidence interval of the inactivation time. The link to download this spreadsheet is available.

As far as we are aware, this is the first report of the thermal inactivation of ASFV in animal and non-animal origin feed ingredients. The heat resistance in terms of *D*_60_, *D*_70_, *D*_80_, and *D*_85_ fulfilled the need of the *D*_T_ of ASFV in feed ingredients, particularly to estimate the probability of at least one ASFV-contaminated vessel of feed ingredients entering an importing country^12^. Such probability is part of the quantitative risk assessment model developed to evaluate the risk of ASFV entry into ASFV-free countries through imported feed ingredients. The outcome of the risk assessment is to initiate risk mitigation measures to minimize the risk of the introduction of ASFV as a transboundary pathogen along the feed supply chain, corresponding to feed biosecurity.

## Conclusion

The thermal inactivation of ASFV in animal and non-animal origin feed ingredients was investigated. The effective inactivation temperatures were as low as 60 °C. The rate of thermal inactivation was represented by *D*_T_ or the time required to reduce ASFV per 1 log at an inactivation temperature (*T*). The mean *D*_60_, *D*_70_, *D*_80_, and *D*_85_ MBM, SBM, and MZ were in the ranges 5.11–6.78, 2.19–3.01, 0.99–2.02, and 0.16–0.99 min, respectively. The heat resistance of ASFV at 60–80 °C in MBM, SBM, and MZ is similar. The *D*_*T*_ models for MBM, SBM, and MZ are log *D*_T_ = − $\left( {\frac{T}{32.08}} \right)$ + 2.69, log *D*_T_ = − $\left( {\frac{T}{31.77}} \right)$ + 2.55, and log *D*_T_ = − $\left( {\frac{T}{18.96}} \right)$ + 4.01 to predict *D*_T_ of the inactivation temperature. a spreadsheet predicting the *D*_T_ and the inactivation time (with 95% confidence interval) from these *D*_T_ models is available to download. The *D*_T_ of ASFV in feed ingredients was used to estimate the probability of contaminated feed ingredients entering an importing country and to evaluate the risk of ASFV entry into ASFV-free countries through imported feed ingredients.

## Supplementary Information

Supplementary Information.

## Data Availability

The spreadsheet supporting the conclusions of this article is available in the https://doi.org/10.6084/m9.figshare.19697185.
